# 2-[2-(3-Chloro­phen­yl)-2-oxoeth­yl]-4-hy­droxy-3-(3-meth­oxy­benzo­yl)-2*H*-1λ^6^,2-benzothia­zine-1,1-dione

**DOI:** 10.1107/S1600536812009014

**Published:** 2012-03-07

**Authors:** Hamid Latif Siddiqui, Matloob Ahmad, Salman Gul, Waseeq Ahmad Siddiqui, Masood Parvez

**Affiliations:** a Institute of Chemistry, University of the Punjab, Lahore 54590, Pakistan; b Chemistry Department, Govt. College University, Faisalabad, Pakistan; c Chemistry Department, University of Sargodha, Sargodha 40100, Pakistan; d Department of Chemistry, The University of Calgary, 2500 University Drive NW, Calgary, Alberta, Canada T2N 1N4

## Abstract

In the title mol­ecule, C_24_H_18_ClNO_6_S, the heterocyclic thia­zine ring adopts a half chair conformation with the S and N atoms displaced by 0.318 (3) and 0.387 (3) Å, respectively, on the opposite sides from the mean plane formed by the remaining ring atoms. The benzene rings of the benzothia­zin unit and meth­oxy­benzoyl group are more or less coplanar, the dihedral angle between the mean planes of these rings being 12.37 (10)° while the chloro­phenyl ring is inclined at 81.87 (4) and 73.30 (5)°, respectively, to these rings. The mol­ecular structure is consolidated by intra­molecular O—H⋯O and C—H⋯N inter­actions and the crystal packing is stabilized by weak inter­molecular C—H⋯O hydrogen bonds.

## Related literature

For background information on the synthesis of related compounds, see: Siddiqui *et al.* (2007 ▶). For the biological activity of benzothia­zine derivatives, see: Turck *et al.* (1995 ▶); Zia-ur-Rehman *et al.* (2006 ▶); Ahmad *et al.* (2010 ▶). For a related structure, see: Siddiqui *et al.* (2008 ▶).
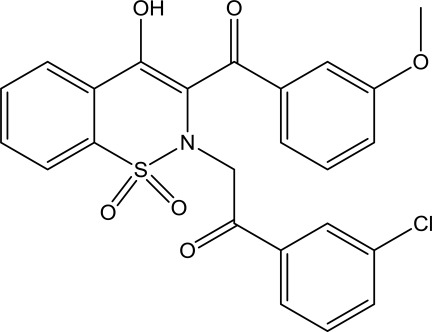


## Experimental

### 

#### Crystal data

C_24_H_18_ClNO_6_S
*M* *_r_* = 483.90
Triclinic, 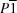

*a* = 10.2562 (3) Å
*b* = 10.9602 (3) Å
*c* = 11.3861 (4) Å
α = 116.5460 (15)°
β = 105.3216 (13)°
γ = 97.2383 (14)°
*V* = 1059.17 (6) Å^3^
*Z* = 2
Mo *K*α radiation
μ = 0.32 mm^−1^
*T* = 173 K
0.16 × 0.10 × 0.08 mm

#### Data collection

Nonius KappaCCD diffractometer
Absorption correction: multi-scan (*SORTAV*; Blessing, 1997 ▶) *T*_min_ = 0.950, *T*_max_ = 0.975
8854 measured reflections
4806 independent reflections
4317 reflections with *I* > 2σ(*I*)
*R*_int_ = 0.022

#### Refinement

*R*[*F* ^2^ > 2σ(*F* ^2^)] = 0.038
*wR*(*F* ^2^) = 0.102
*S* = 1.04
4806 reflections
300 parameters
H-atom parameters constrained
Δρ_max_ = 0.36 e Å^−3^
Δρ_min_ = −0.44 e Å^−3^

### 

Data collection: *COLLECT* (Hooft, 1998 ▶); cell refinement: *DENZO* (Otwinowski & Minor, 1997 ▶); data reduction: *SCALEPACK* (Otwinowski & Minor, 1997 ▶); program(s) used to solve structure: *SHELXS97* (Sheldrick, 2008 ▶); program(s) used to refine structure: *SHELXL97* (Sheldrick, 2008 ▶); molecular graphics: *ORTEP-3 for Windows* (Farrugia, 1997 ▶); software used to prepare material for publication: *SHELXL97*.

## Supplementary Material

Crystal structure: contains datablock(s) global, I. DOI: 10.1107/S1600536812009014/pk2394sup1.cif

Structure factors: contains datablock(s) I. DOI: 10.1107/S1600536812009014/pk2394Isup2.hkl

Supplementary material file. DOI: 10.1107/S1600536812009014/pk2394Isup3.cml

Additional supplementary materials:  crystallographic information; 3D view; checkCIF report

## Figures and Tables

**Table 1:** Hydrogen-bond geometry (Å, °)

*D*—H⋯*A*	*D*—H	H⋯*A*	*D*⋯*A*	*D*—H⋯*A*
C4—H4⋯O5^i^	0.95	2.49	3.365 (2)	154
C17—H17*A*⋯O2^ii^	0.99	2.26	3.2467 (19)	174
O3—H3*O*⋯O4	0.84	1.70	2.4528 (18)	148
C11—H11⋯N1	0.95	2.40	2.972 (2)	118

Symmetry codes: (i) 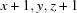; (ii) 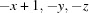.

**Fig. 1.**
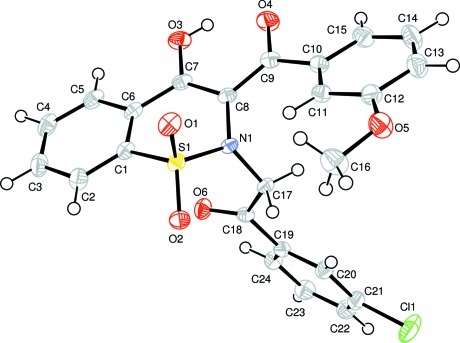
The molecular structure of the title compound. Displacement ellipsoids are drawn at the 50% probability level. H atoms are presented as small spheres of arbitrary radius.

**Fig. 2.**
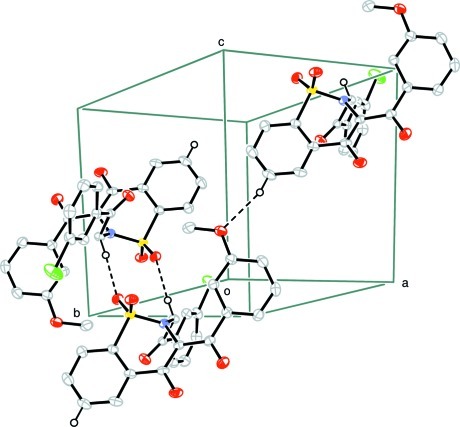
A view of the C—-H···O hydrogen bonds (dotted lines) in the crystal structure of the title compound. H atoms not participating in hydrogen- bonding have been omitted for clarity.

## supplementary crystallographic information

### Comment

Derivatives of benzothiazine have been studied for a broad range of biological activities. They are found to possess analgesic (Turck *et al*., 1995), antimicrobial (Zia-ur-Rehman *et al.*, 2006) and antioxidant activities (Ahmad *et al.*, 2010), *etc*. In continuation of our research on the synthesis of biologically active benzothiazine derivatives (Siddiqui *et al.*, 2007; Ahmad *et al.*, 2010), we herein report the synthesis and crystal structure of the title compound.

The bond distances and angles (Fig. 1) agree very well with the corresponding bond distances and angles reported in closely related compounds (Siddiqui *et al.*, 2008). The heterocyclic thiazine ring adopts a half chair conformation with atoms N1 and S1 displaced by 0.387 (3) and 0.318 (3) Å, respectively, on the opposite sides from the mean plane formed by the remaining ring atoms. The benzene rings C1–C6 and C10–C15 are more or less co-planar with a dihedral angle between the mean planes of these rings being 12.37 (10)°; the benzene ring C19–C24 is oriented at 81.87 (4) and 73.30 (5)°, respectively, with respect to these benzene rings. While the molecular structure of the title compound is consolidated by intramolecular interactions: O3–H3O···O4 and C11–H11···N1, the crystal packing is stabilized by weak intermolecular C—H···O hydrogen bonds (Fig. 2 and Table 1).

### Experimental

A mixture of (4-hydroxy-1,1-dioxido-2*H*-1,2-benzothiazin-3-yl)(3-methoxyphenyl) methanone (5.0 g, 0.015 mol), K_2_CO_3_ (2.07 g, 0.015 mol) and 3-chlorophenacyl bromide (3.50 g, 0.015 mol) in acetonitrile (30 ml) was refluxed for 3 h. The contents of the flask were poured on ice cold HCl (5%, 30 ml). The precipitates of the title compound thus formed were collected and washed with ethanol. The crystals suitable for X-ray crystallographic analysis were grown from a solution in methanol.

### Refinement

All H atoms were positioned geometrically and refined using a riding model, with O—H = 0.84 Å and C—H = 0.95, 0.98 and 0.99 Å, for aryl, methyl and methylene H-atoms, respectively. The *U*_iso_(H) were allowed at 1.5*U*_eq_(O) or 1.2*U*_eq_(C).

### Crystal data

**Table:**

C_24_H_18_ClNO_6_S	*Z* = 2
*M**_r_* = 483.90	*F*(000) = 500
Triclinic, *P*1	*D*_x_ = 1.517 Mg m^−^^3^
Hall symbol: -P 1	Mo *K*α radiation, λ = 0.71073 Å
*a* = 10.2562 (3) Å	Cell parameters from 4529 reflections
*b* = 10.9602 (3) Å	θ = 1.0–27.5°
*c* = 11.3861 (4) Å	µ = 0.32 mm^−^^1^
α = 116.5460 (15)°	*T* = 173 K
β = 105.3216 (13)°	Prism, yellow
γ = 97.2383 (14)°	0.16 × 0.10 × 0.08 mm
*V* = 1059.17 (6) Å^3^	

### Data collection

**Table:**

Nonius KappaCCD diffractometer	4806 independent reflections
Radiation source: fine-focus sealed tube	4317 reflections with *I* > 2σ(*I*)
Graphite monochromator	*R*_int_ = 0.022
ω and φ scans	θ_max_ = 27.6°, θ_min_ = 2.2°
Absorption correction: multi-scan (*SORTAV*; Blessing, 1997)	*h* = −13→13
*T*_min_ = 0.950, *T*_max_ = 0.975	*k* = −14→13
8854 measured reflections	*l* = −14→14

### Refinement

**Table:**

Refinement on *F*^2^	Primary atom site location: structure-invariant direct methods
Least-squares matrix: full	Secondary atom site location: difference Fourier map
*R*[*F*^2^ > 2σ(*F*^2^)] = 0.038	Hydrogen site location: inferred from neighbouring sites
*wR*(*F*^2^) = 0.102	H-atom parameters constrained
*S* = 1.04	*w* = 1/[σ^2^(*F*_o_^2^) + (0.0427*P*)^2^ + 0.7415*P*] where *P* = (*F*_o_^2^ + 2*F*_c_^2^)/3
4806 reflections	(Δ/σ)_max_ = 0.001
300 parameters	Δρ_max_ = 0.36 e Å^−^^3^
0 restraints	Δρ_min_ = −0.44 e Å^−^^3^

### Special details

**Table:**

Geometry. All e.s.d.'s (except the e.s.d. in the dihedral angle between two l.s. planes) are estimated using the full covariance matrix. The cell e.s.d.'s are taken into account individually in the estimation of e.s.d.'s in distances, angles and torsion angles; correlations between e.s.d.'s in cell parameters are only used when they are defined by crystal symmetry. An approximate (isotropic) treatment of cell e.s.d.'s is used for estimating e.s.d.'s involving l.s. planes.
Refinement. Refinement of *F*^2^ against ALL reflections. The weighted *R*-factor *wR* and goodness of fit *S* are based on *F*^2^, conventional *R*-factors *R* are based on *F*, with *F* set to zero for negative *F*^2^. The threshold expression of *F*^2^ > σ(*F*^2^) is used only for calculating *R*-factors(gt) *etc*. and is not relevant to the choice of reflections for refinement. *R*-factors based on *F*^2^ are statistically about twice as large as those based on *F*, and *R*- factors based on ALL data will be even larger.

### Fractional atomic coordinates and isotropic or equivalent isotropic displacement parameters (Å^2^)

**Table:**

	*x*	*y*	*z*	*U*_iso_*/*U*_eq_	
Cl1	−0.05797 (5)	−0.18324 (6)	−0.01778 (6)	0.04844 (16)	
S1	0.71725 (4)	0.22320 (4)	0.20290 (4)	0.01987 (10)	
O1	0.77653 (13)	0.30923 (13)	0.15492 (12)	0.0277 (3)	
O2	0.66169 (12)	0.07248 (12)	0.10925 (12)	0.0263 (3)	
O3	0.79628 (14)	0.59012 (13)	0.60161 (12)	0.0295 (3)	
H3O	0.7398	0.6368	0.5906	0.044*	
O4	0.59391 (14)	0.64360 (13)	0.48266 (13)	0.0329 (3)	
O5	0.30836 (15)	0.31540 (14)	−0.14637 (13)	0.0341 (3)	
O6	0.57415 (12)	0.12424 (13)	0.38675 (13)	0.0268 (3)	
N1	0.59256 (13)	0.28253 (13)	0.25358 (13)	0.0183 (3)	
C1	0.84360 (16)	0.25902 (17)	0.36151 (16)	0.0216 (3)	
C2	0.93526 (18)	0.17556 (19)	0.35821 (19)	0.0285 (4)	
H2	0.9244	0.0936	0.2728	0.034*	
C3	1.04362 (18)	0.2150 (2)	0.4833 (2)	0.0321 (4)	
H3	1.1077	0.1595	0.4834	0.038*	
C4	1.05862 (18)	0.3346 (2)	0.60758 (19)	0.0302 (4)	
H4	1.1346	0.3620	0.6915	0.036*	
C5	0.96382 (17)	0.41500 (19)	0.61074 (17)	0.0263 (3)	
H5	0.9735	0.4953	0.6970	0.032*	
C6	0.85398 (16)	0.37743 (17)	0.48665 (16)	0.0216 (3)	
C7	0.75688 (17)	0.46543 (16)	0.48731 (16)	0.0217 (3)	
C8	0.63586 (16)	0.42400 (16)	0.37133 (16)	0.0199 (3)	
C9	0.56134 (17)	0.52651 (17)	0.36927 (17)	0.0231 (3)	
C10	0.44896 (17)	0.50693 (17)	0.24294 (17)	0.0227 (3)	
C11	0.43680 (17)	0.41371 (17)	0.10442 (17)	0.0223 (3)	
H11	0.4999	0.3570	0.0873	0.027*	
C12	0.33198 (18)	0.40499 (18)	−0.00728 (18)	0.0259 (3)	
C13	0.2411 (2)	0.4903 (2)	0.0180 (2)	0.0343 (4)	
H13	0.1704	0.4853	−0.0587	0.041*	
C14	0.2539 (2)	0.5820 (2)	0.1544 (2)	0.0368 (4)	
H14	0.1914	0.6395	0.1710	0.044*	
C15	0.35727 (19)	0.59146 (19)	0.26782 (19)	0.0296 (4)	
H15	0.3654	0.6549	0.3615	0.036*	
C16	0.3904 (2)	0.2167 (2)	−0.1782 (2)	0.0379 (4)	
H16A	0.3618	0.1575	−0.2805	0.046*	
H16B	0.3751	0.1560	−0.1379	0.046*	
H16C	0.4905	0.2690	−0.1378	0.046*	
C17	0.45869 (15)	0.18377 (16)	0.21602 (16)	0.0192 (3)	
H17A	0.4274	0.1099	0.1155	0.023*	
H17B	0.3870	0.2364	0.2268	0.023*	
C18	0.46425 (16)	0.11098 (16)	0.30312 (16)	0.0195 (3)	
C19	0.32618 (16)	0.02199 (16)	0.27953 (17)	0.0210 (3)	
C20	0.21055 (17)	−0.03201 (17)	0.15516 (18)	0.0242 (3)	
H20	0.2179	−0.0139	0.0826	0.029*	
C21	0.08465 (17)	−0.11260 (18)	0.13939 (19)	0.0277 (4)	
C22	0.07093 (19)	−0.13897 (19)	0.2444 (2)	0.0302 (4)	
H22	−0.0167	−0.1923	0.2327	0.036*	
C23	0.18708 (19)	−0.08632 (19)	0.3669 (2)	0.0300 (4)	
H23	0.1793	−0.1051	0.4389	0.036*	
C24	0.31434 (18)	−0.00654 (18)	0.38496 (18)	0.0248 (3)	
H24	0.3935	0.0287	0.4689	0.030*	

### Atomic displacement parameters (Å^2^)

**Table:**

	*U*^11^	*U*^22^	*U*^33^	*U*^12^	*U*^13^	*U*^23^
Cl1	0.0257 (2)	0.0561 (3)	0.0516 (3)	−0.0096 (2)	−0.0072 (2)	0.0346 (3)
S1	0.01920 (18)	0.02034 (19)	0.01508 (18)	0.00331 (14)	0.00465 (14)	0.00631 (15)
O1	0.0276 (6)	0.0330 (6)	0.0224 (6)	0.0040 (5)	0.0101 (5)	0.0144 (5)
O2	0.0265 (6)	0.0205 (6)	0.0208 (6)	0.0059 (5)	0.0054 (5)	0.0035 (5)
O3	0.0374 (7)	0.0214 (6)	0.0169 (6)	0.0031 (5)	0.0036 (5)	0.0042 (5)
O4	0.0382 (7)	0.0225 (6)	0.0241 (6)	0.0097 (5)	0.0073 (5)	0.0026 (5)
O5	0.0408 (7)	0.0355 (7)	0.0219 (6)	0.0151 (6)	0.0058 (5)	0.0132 (6)
O6	0.0212 (6)	0.0326 (6)	0.0267 (6)	0.0057 (5)	0.0043 (5)	0.0178 (5)
N1	0.0173 (6)	0.0158 (6)	0.0161 (6)	0.0012 (5)	0.0040 (5)	0.0055 (5)
C1	0.0187 (7)	0.0235 (7)	0.0194 (7)	0.0021 (6)	0.0046 (6)	0.0105 (6)
C2	0.0238 (8)	0.0319 (9)	0.0285 (9)	0.0084 (7)	0.0081 (7)	0.0147 (7)
C3	0.0210 (8)	0.0415 (10)	0.0386 (10)	0.0096 (7)	0.0074 (7)	0.0257 (9)
C4	0.0207 (8)	0.0407 (10)	0.0277 (9)	0.0006 (7)	0.0015 (7)	0.0219 (8)
C5	0.0241 (8)	0.0296 (8)	0.0193 (7)	−0.0029 (6)	0.0018 (6)	0.0135 (7)
C6	0.0195 (7)	0.0222 (7)	0.0184 (7)	−0.0016 (6)	0.0033 (6)	0.0103 (6)
C7	0.0248 (8)	0.0193 (7)	0.0156 (7)	−0.0004 (6)	0.0052 (6)	0.0073 (6)
C8	0.0220 (7)	0.0170 (7)	0.0162 (7)	0.0020 (6)	0.0067 (6)	0.0059 (6)
C9	0.0261 (8)	0.0195 (7)	0.0215 (8)	0.0040 (6)	0.0095 (7)	0.0087 (6)
C10	0.0248 (8)	0.0208 (7)	0.0244 (8)	0.0058 (6)	0.0095 (7)	0.0126 (7)
C11	0.0244 (8)	0.0209 (7)	0.0227 (8)	0.0064 (6)	0.0087 (6)	0.0118 (6)
C12	0.0283 (8)	0.0248 (8)	0.0237 (8)	0.0062 (6)	0.0071 (7)	0.0129 (7)
C13	0.0343 (10)	0.0365 (10)	0.0329 (10)	0.0148 (8)	0.0072 (8)	0.0197 (8)
C14	0.0359 (10)	0.0378 (10)	0.0414 (11)	0.0222 (8)	0.0149 (9)	0.0201 (9)
C15	0.0338 (9)	0.0271 (8)	0.0284 (9)	0.0116 (7)	0.0132 (7)	0.0126 (7)
C16	0.0508 (12)	0.0354 (10)	0.0245 (9)	0.0187 (9)	0.0131 (9)	0.0112 (8)
C17	0.0167 (7)	0.0179 (7)	0.0170 (7)	0.0002 (5)	0.0023 (6)	0.0073 (6)
C18	0.0229 (7)	0.0161 (7)	0.0173 (7)	0.0051 (6)	0.0076 (6)	0.0064 (6)
C19	0.0211 (7)	0.0182 (7)	0.0230 (8)	0.0050 (6)	0.0079 (6)	0.0099 (6)
C20	0.0228 (8)	0.0243 (8)	0.0250 (8)	0.0046 (6)	0.0070 (6)	0.0133 (7)
C21	0.0202 (8)	0.0265 (8)	0.0340 (9)	0.0034 (6)	0.0051 (7)	0.0165 (7)
C22	0.0264 (8)	0.0274 (9)	0.0418 (10)	0.0064 (7)	0.0159 (8)	0.0197 (8)
C23	0.0332 (9)	0.0310 (9)	0.0327 (9)	0.0084 (7)	0.0161 (8)	0.0193 (8)
C24	0.0261 (8)	0.0255 (8)	0.0242 (8)	0.0077 (6)	0.0096 (7)	0.0134 (7)

### Geometric parameters (Å, º)

**Table:**

Cl1—C21	1.7419 (18)	C10—C15	1.396 (2)
S1—O1	1.4312 (12)	C10—C11	1.404 (2)
S1—O2	1.4345 (12)	C11—C12	1.388 (2)
S1—N1	1.6295 (13)	C11—H11	0.9500
S1—C1	1.7596 (16)	C12—C13	1.395 (2)
O3—C7	1.3107 (19)	C13—C14	1.380 (3)
O3—H3O	0.8400	C13—H13	0.9500
O4—C9	1.268 (2)	C14—C15	1.390 (3)
O5—C12	1.370 (2)	C14—H14	0.9500
O5—C16	1.432 (2)	C15—H15	0.9500
O6—C18	1.2109 (19)	C16—H16A	0.9800
N1—C8	1.4327 (19)	C16—H16B	0.9800
N1—C17	1.4632 (18)	C16—H16C	0.9800
C1—C2	1.388 (2)	C17—C18	1.521 (2)
C1—C6	1.399 (2)	C17—H17A	0.9900
C2—C3	1.394 (2)	C17—H17B	0.9900
C2—H2	0.9500	C18—C19	1.499 (2)
C3—C4	1.386 (3)	C19—C20	1.396 (2)
C3—H3	0.9500	C19—C24	1.399 (2)
C4—C5	1.389 (3)	C20—C21	1.388 (2)
C4—H4	0.9500	C20—H20	0.9500
C5—C6	1.399 (2)	C21—C22	1.386 (3)
C5—H5	0.9500	C22—C23	1.388 (3)
C6—C7	1.470 (2)	C22—H22	0.9500
C7—C8	1.395 (2)	C23—C24	1.386 (2)
C8—C9	1.441 (2)	C23—H23	0.9500
C9—C10	1.493 (2)	C24—H24	0.9500
			
O1—S1—O2	118.90 (7)	O5—C12—C13	115.33 (15)
O1—S1—N1	108.48 (7)	C11—C12—C13	120.23 (16)
O2—S1—N1	107.88 (7)	C14—C13—C12	119.94 (17)
O1—S1—C1	108.06 (7)	C14—C13—H13	120.0
O2—S1—C1	109.85 (7)	C12—C13—H13	120.0
N1—S1—C1	102.41 (7)	C13—C14—C15	120.79 (17)
C7—O3—H3O	109.5	C13—C14—H14	119.6
C12—O5—C16	117.54 (14)	C15—C14—H14	119.6
C8—N1—C17	120.09 (12)	C14—C15—C10	119.41 (16)
C8—N1—S1	116.00 (10)	C14—C15—H15	120.3
C17—N1—S1	120.54 (10)	C10—C15—H15	120.3
C2—C1—C6	122.05 (15)	O5—C16—H16A	109.5
C2—C1—S1	119.52 (13)	O5—C16—H16B	109.5
C6—C1—S1	118.30 (12)	H16A—C16—H16B	109.5
C1—C2—C3	118.36 (17)	O5—C16—H16C	109.5
C1—C2—H2	120.8	H16A—C16—H16C	109.5
C3—C2—H2	120.8	H16B—C16—H16C	109.5
C4—C3—C2	120.50 (17)	N1—C17—C18	114.55 (12)
C4—C3—H3	119.8	N1—C17—H17A	108.6
C2—C3—H3	119.8	C18—C17—H17A	108.6
C3—C4—C5	120.73 (16)	N1—C17—H17B	108.6
C3—C4—H4	119.6	C18—C17—H17B	108.6
C5—C4—H4	119.6	H17A—C17—H17B	107.6
C4—C5—C6	119.85 (16)	O6—C18—C19	121.96 (14)
C4—C5—H5	120.1	O6—C18—C17	121.81 (14)
C6—C5—H5	120.1	C19—C18—C17	116.24 (13)
C5—C6—C1	118.44 (15)	C20—C19—C24	120.00 (14)
C5—C6—C7	120.38 (15)	C20—C19—C18	121.19 (14)
C1—C6—C7	121.10 (14)	C24—C19—C18	118.81 (14)
O3—C7—C8	121.86 (15)	C21—C20—C19	118.81 (15)
O3—C7—C6	115.32 (14)	C21—C20—H20	120.6
C8—C7—C6	122.69 (14)	C19—C20—H20	120.6
C7—C8—N1	119.22 (14)	C22—C21—C20	121.65 (16)
C7—C8—C9	119.34 (14)	C22—C21—Cl1	119.77 (13)
N1—C8—C9	121.35 (14)	C20—C21—Cl1	118.57 (14)
O4—C9—C8	118.08 (15)	C21—C22—C23	119.06 (16)
O4—C9—C10	116.78 (14)	C21—C22—H22	120.5
C8—C9—C10	125.13 (14)	C23—C22—H22	120.5
C15—C10—C11	120.10 (15)	C24—C23—C22	120.50 (16)
C15—C10—C9	116.76 (15)	C24—C23—H23	119.8
C11—C10—C9	123.08 (14)	C22—C23—H23	119.8
C12—C11—C10	119.52 (15)	C23—C24—C19	119.96 (16)
C12—C11—H11	120.2	C23—C24—H24	120.0
C10—C11—H11	120.2	C19—C24—H24	120.0
O5—C12—C11	124.44 (15)		
			
O1—S1—N1—C8	−63.67 (12)	C7—C8—C9—C10	167.21 (14)
O2—S1—N1—C8	166.30 (11)	N1—C8—C9—C10	−9.4 (2)
C1—S1—N1—C8	50.42 (12)	O4—C9—C10—C15	−21.7 (2)
O1—S1—N1—C17	137.03 (11)	C8—C9—C10—C15	159.13 (16)
O2—S1—N1—C17	7.00 (14)	O4—C9—C10—C11	155.54 (15)
C1—S1—N1—C17	−108.87 (12)	C8—C9—C10—C11	−23.6 (2)
O1—S1—C1—C2	−92.59 (14)	C15—C10—C11—C12	−0.8 (2)
O2—S1—C1—C2	38.57 (15)	C9—C10—C11—C12	−177.97 (15)
N1—S1—C1—C2	153.01 (13)	C16—O5—C12—C11	5.0 (3)
O1—S1—C1—C6	83.31 (14)	C16—O5—C12—C13	−174.59 (17)
O2—S1—C1—C6	−145.53 (12)	C10—C11—C12—O5	−178.35 (15)
N1—S1—C1—C6	−31.09 (14)	C10—C11—C12—C13	1.3 (2)
C6—C1—C2—C3	−2.3 (2)	O5—C12—C13—C14	178.59 (17)
S1—C1—C2—C3	173.42 (13)	C11—C12—C13—C14	−1.1 (3)
C1—C2—C3—C4	0.1 (3)	C12—C13—C14—C15	0.4 (3)
C2—C3—C4—C5	1.9 (3)	C13—C14—C15—C10	0.0 (3)
C3—C4—C5—C6	−1.7 (2)	C11—C10—C15—C14	0.2 (3)
C4—C5—C6—C1	−0.4 (2)	C9—C10—C15—C14	177.50 (16)
C4—C5—C6—C7	−177.14 (15)	C8—N1—C17—C18	−81.30 (17)
C2—C1—C6—C5	2.5 (2)	S1—N1—C17—C18	77.15 (15)
S1—C1—C6—C5	−173.33 (12)	N1—C17—C18—O6	−6.8 (2)
C2—C1—C6—C7	179.16 (15)	N1—C17—C18—C19	173.22 (12)
S1—C1—C6—C7	3.4 (2)	O6—C18—C19—C20	−158.08 (15)
C5—C6—C7—O3	12.2 (2)	C17—C18—C19—C20	21.9 (2)
C1—C6—C7—O3	−164.46 (14)	O6—C18—C19—C24	22.0 (2)
C5—C6—C7—C8	−171.91 (14)	C17—C18—C19—C24	−158.04 (14)
C1—C6—C7—C8	11.4 (2)	C24—C19—C20—C21	0.6 (2)
O3—C7—C8—N1	−175.40 (14)	C18—C19—C20—C21	−179.32 (14)
C6—C7—C8—N1	9.0 (2)	C19—C20—C21—C22	0.8 (3)
O3—C7—C8—C9	7.9 (2)	C19—C20—C21—Cl1	−178.48 (12)
C6—C7—C8—C9	−167.76 (14)	C20—C21—C22—C23	−1.6 (3)
C17—N1—C8—C7	115.68 (16)	Cl1—C21—C22—C23	177.64 (14)
S1—N1—C8—C7	−43.71 (17)	C21—C22—C23—C24	1.0 (3)
C17—N1—C8—C9	−67.67 (19)	C22—C23—C24—C19	0.3 (3)
S1—N1—C8—C9	132.94 (13)	C20—C19—C24—C23	−1.1 (2)
C7—C8—C9—O4	−12.0 (2)	C18—C19—C24—C23	178.79 (15)
N1—C8—C9—O4	171.39 (14)		

### Hydrogen-bond geometry (Å, º)

**Table:**

*D*—H···*A*	*D*—H	H···*A*	*D*···*A*	*D*—H···*A*
C4—H4···O5^i^	0.95	2.49	3.365 (2)	154
C17—H17*A*···O2^ii^	0.99	2.26	3.2467 (19)	174
O3—H3*O*···O4	0.84	1.70	2.4528 (18)	148
C11—H11···N1	0.95	2.40	2.972 (2)	118
C17—H17*A*···O2	0.99	2.50	2.8199 (19)	98

Symmetry codes: (i) *x*+1, *y*, *z*+1; (ii) −*x*+1, −*y*, −*z*.
